# Construction Notes

**Published:** 1893-07-29

**Authors:** 


### CONSTRUCTION NOTES.

The Fever Hospital, Wallsend. — The finishing touches have now been given to the new infectious diseases hospital at Wallsend, erected in accordance with a mutual agreement between the joint authorities of Wallsend, Willington Quay, and Howden, of the Mid-Tyne district. The area covers two acres, and the erections within the enclosure comprise four unattached blocks of buildings, the front, or western entrance, rising to a height of three storeys. Accommodation is provided in this wing for the resident surgeon, matron, porters, nurses, and attendants, together with a large kitchen and sculleries on the ground floor. The south block is built in the pavilion style of architecture, and is divided into two isolated wings, for the treatment of any two distinct classes of infectious diseases. Six beds are provided in the ward, and all the apartments are lofty and well lighted, while in the ventilation arrangements all the latest improvements have been introduced. The east wing consists of spacious laundries and wash-houses, as also including a disinfecting store. The north block contains six beds for males and six for females, in addition to apartments specially set apart for the use of nurses. A mortuary and an ambulance have been provided within the enclosure. The accommodation now provides eighteen beds for the use of patients, and provision is made for increasing the number to thirty. The main entrance wing is in three divisions, comprising bath and dressing rooms and disinfecting-rooms for the patients. The contract for the building was accepted from Mr. Nicol Ritchie, of Whitley, the architectural plans being designed by Mr. William Hope, of North Shields and Newcastle.

